# Influence of Rearing Systems on the Genetic, Nutritional and Medicinal Properties of Edible Insects: A Critical Review

**DOI:** 10.3390/foods15213858

**Published:** 2026-10-28

**Authors:** Fègbawè Badanaro, Séverin Tchibozo, Victor Benno Meyer-Rochow

**Affiliations:** 1 Département de Biochimie, Faculté des Sciences, Université de Lomé, Lomé P.O. Box 1515, Togo; 2 Centre de Recherche pour la Gestion de la Biodiversité (CRGB), Cotonou 04 B.P. 0385, Benin; tchisev@yahoo.fr; 3 Department of Ecology and Genetics, University of Oulu, 90570 Oulu, Finland

**Keywords:** edible insect rearing, rearing conditions, gene expression, genetic diversity, nutritional quality, medicinal properties, human consumption

## Abstract

Insect farming is gaining momentum for its potential to strengthen food security and the sustainability of food systems. However, how rearing conditions shape the overall quality of edible insects remains poorly understood. Optimization strategies are suggested on the basis of this critical review, which synthesizes 96 studies, selected from 866 database records and supplemented by citation searching, on the effects of rearing systems and genetic, nutritional, and medicinal properties of edible insects. At least 41 species are farmed under conventional, semi-intensive, or intensive systems, and their quality behaves as a plastic rather than intrinsic trait, modulated by nutritional, environmental, husbandry-related, and biological factors acting on both phenotype (plasticity, epigenetic regulation) and genotype (drift, inbreeding, selection). Reported effects are substantial. For instance, in *Tenebrio molitor*, crude protein falls from 64.3 to 54.4 g/100 g dry matter as temperature rises from 22 to 28 °C, lipid content peaks at 47.4% at 31 °C, and artificial selection has raised larval weight by up to 39% within 16 generations. Conventional and semi-intensive systems show high batch-to-batch and seasonal variability, whereas intensive systems deliver more stable, standardized production at higher cost and lower consumer acceptability. Genetic selection emerges as a promising but underexploited lever, although conclusions remain qualified by study heterogeneity.

## 1. Introduction

The Food and Agriculture Organization has called for the development of sustainable alternative food sources, among which edible insects, first suggested by Meyer-Rochow [1], occupy an increasingly prominent place [2]. Edible insects are highly nutritious, providing abundant high-quality protein that contains all the essential amino acids, lipids rich in unsaturated fatty acids, and essential micronutrients such as iron, zinc, and magnesium [3,4,5,6,7]. Beyond their nutritional value, several species are recognized for medicinal properties documented in a growing body of research [8,9,10]. For many human populations, as well as animals such as fish, amphibians, reptiles, birds, and mammals, insects have, and continue to play, an important role as a food item. In connection with human health, insects, moreover, can be used curatively or preventively, directly or indirectly through ingestion [11,12]. Their production also offers clear environmental advantages over conventional livestock: a low ecological footprint, with lower land and water requirements and reduced greenhouse gas and ammonia emissions [13,14,15].

Insects can be reared on a range of decomposing organic substrates, such as compost or manure, thereby valorizing organic waste [2]. They further display high reproductive rates, good disease resistance, strong adaptability to diverse ecosystems, and relatively short life cycles [16,17]. Occupying low trophic levels, they exploit a wider diversity of plant resources than conventional livestock and reproduce rapidly in large numbers [18]. As ectotherms, they convert ingested feed into biomass with high efficiency, giving them an excellent feed conversion ratio [19,20].

The nutritional, environmental, and medicinal potential of edible insects has attracted growing scientific and industrial interest over recent decades, driving the rapid expansion of insect rearing worldwide ever since Kok et al. [21] described the design and operation of an insect farm. This growth responds to the rising demand for sustainable alternative protein and to the need to diversify food production in the face of climate change, resource depletion, and global population growth, even though populations are declining in many European and Asian countries [22,23]. As a result, optimizing rearing practices has become a priority for achieving stable, safe, and high-quality production aligned with the nutritional, sanitary, and technological requirements of emerging markets.

Despite this progress, several scientific challenges remain. Chief among them, the influence of rearing conditions on the overall quality of edible insects is still insufficiently understood. Factors such as feed substrate, population density, temperature, relative humidity, and photoperiod, together with selection and breeding practices, can substantially modulate the biological and biochemical traits of reared insects in both males and females [24,25,26]. Several studies show that rearing practices affect insect physiology and phenotype, including growth, survival, and feed conversion efficiency [16,27,28,29]. Such phenotypic variation may arise from epigenetic mechanisms that modulate gene expression without altering the DNA sequence and are therefore reversible, depending on the rearing conditions [30,31].

Additionally, certain practices (selection direction in particular) can acutely reshape the genetic structure of populations by favoring specific genotypes associated with superior performance [32,33]. Even without intentional selection, maintaining insects under confined conditions can drive unintended genetic changes such as drift, founder effects, and inbreeding depression [34,35], progressively separating reared from wild populations. These phenotypic and genotypic changes in turn influence nutritional composition [28,36,37] and may modulate medicinal properties and pharmaceutical effectiveness [8,38]. These dimensions are thus tightly interconnected, underscoring the need for a systemic approach to assess the overall impact of rearing practices.

Although several parameters have been examined in various species of economic interest, the available knowledge remains fragmented and often confined to isolated aspects, lacking an integrative view of how rearing conditions and edible insect quality interact. A critical synthesis of the evidence is therefore needed to characterize the effects of rearing systems across the different dimensions of quality and to guide future research and optimization for food and medicinal use. This study critically reviews the literature on the impact of rearing conditions on the genetic, nutritional, and medicinal properties of edible insects, and identifies optimization strategies for food and medicinal uses.

## 2. Review Methodology

### 2.1. Databases Consulted

**Figure 1:**
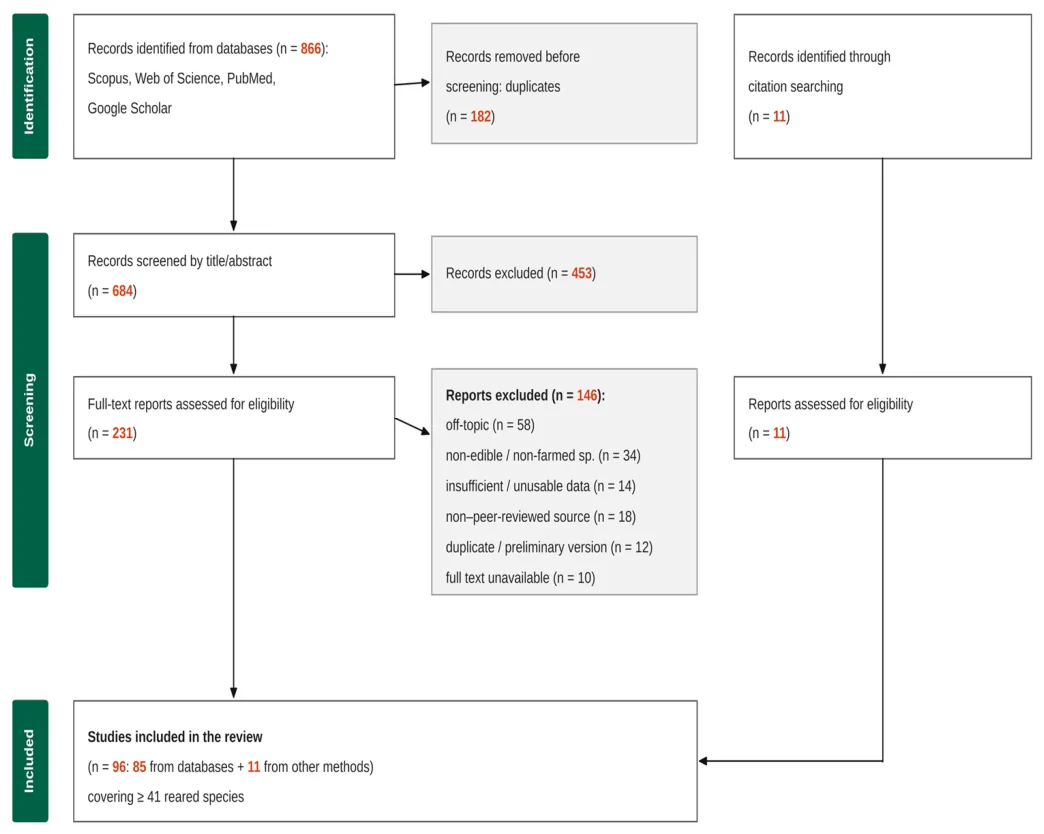
Study selection flow diagram (database search and other methods).

The literature search drew on four major scientific databases (Scopus, Web of Science, PubMed, and Google Scholar) and returned 866 records (Figure 1). After removing 182 duplicates, 684 articles were screened by title and abstract, and 453 were excluded. The remaining 231 full-text articles were assessed for eligibility, and 146 were excluded for the following reasons: off-topic (n = 58), non-edible or non-farmed species (n = 34), insufficient or unusable data (n = 14), non-peer-reviewed source (n = 18), duplicate or preliminary version (n = 12), and full text unavailable (n = 10). The resulting 85 studies were retained from the database search. In addition, 11 studies identified through other methods (citation searching) met the same eligibility criteria and were added, so 96 studies formed the core corpus analyzed for the effects of rearing conditions. For contextual and taxonomic purposes, this corpus was supplemented with additional references (scientific books and book chapters, reports from international organizations, and regulatory texts), bringing the total number of works cited to 215. Although the review follows a narrative and critical approach, the selection details are reported to ensure transparency in the identification and screening process.

### 2.2. Search Strategy and Keywords

The search used English-language keywords to maximize retrieval of the international literature. The main terms were: edible insects, medicinal insects, insect farming, insect rearing, rearing conditions, genetic variation, genetic diversity, gene expression, nutritional composition, medicinal properties, bioactive compounds, and pharmacological activities. These were combined with Boolean operators (AND, OR) and supplemented with synonyms and terminological variants (e.g., mass rearing, silkworm, black soldier fly, mealworm, cricket, frass, antimicrobial peptides) to broaden coverage. The search targeted works published between 2000 and 2026, with particular attention to the most recent studies.

### 2.3. Types of Sources and Inclusion and Exclusion Criteria

Given the emerging and cross-disciplinary nature of the field, the review draws primarily on peer-reviewed articles (experimental studies, reviews, and meta-analyses), supplemented, where needed for context, by a limited number of reference sources: scientific books and book chapters, reports from international organizations (notably the FAO) [39], and normative and regulatory texts (EFSA opinions [40] and European Union regulations on novel foods and insect proteins).

Studies were included if they drew on reliable, verifiable scientific or institutional sources; addressed edible or farmed insects (including species domesticated for non-food purposes, such as bees and silkworms, relevant to the species inventory); and reported effects of rearing conditions on genetic diversity, nutritional composition, medicinal properties, or bioactive compounds. Studies were excluded if they consisted of non-scientific or non-verifiable content (blogs, opinion pieces, interviews), provided insufficient or irrelevant data, or were duplicates across databases.

### 2.4. Data Analysis Method

The selected sources underwent a qualitative, comparative analysis. First, relevant information was extracted and organized along the review’s three main axes: (i) the effects of rearing conditions on genetic diversity and trait expression; (ii) their influence on nutritional composition; and (iii) their effects on medicinal and bioactive properties, particularly antioxidant and antimicrobial activities. Particular attention was paid to rearing factors (feed and substrate, temperature, humidity, photoperiod, population density, the genetics of farmed populations, associated microbiota, and reproduction practices) and their effects on the insects’ biological and biochemical traits. Second, a comparative synthesis identified general trends, convergences, and divergences among studies, highlighted interactions among the genetic, nutritional, and medicinal dimensions, and mapped gaps in the literature. Finally, the findings were interpreted from an integrated perspective to propose recommendations for optimizing rearing practices, improving the overall quality of insects intended for consumption, and outlining priorities for future research.

## 3. Overview of Edible Insect Species, Rearing Systems and Rearing Factors

### 3.1. Species Reared in Insect Farming

More than 2205 insect species have been recorded as edible worldwide [41], although there has been some criticism concerning the validity of this number, and Van Itterbeeck and Pelozuelo [42] arrived at a figure of 1611 for food insects. However, farming insects for food and feed relies on a comparatively small number [43,44]. Some species have been domesticated for millennia for non-food purposes, including the silkworm (*Bombyx mori* (Linnaeus, 1758) (Lepidoptera: Bombycidae)) for silk, the honey bee (*Apis mellifera* Linnaeus, 1758 (Hymenoptera: Apidae)) for honey and other bee products, and the cochineal (*Dactylopius coccus* Costa, 1835 (Hemiptera: Dactylopiidae)) for natural dye [45]. Bee larvae, silkworm pupae, and adult cochineal are also valued as nutrient-rich food by-products [41,46].

By contrast, interest in rearing other edible insects specifically for food rather than as feed for pets such as tropical fish, frogs, and lizards is relatively recent and has grown considerably in recent decades [47]. In all, Appendix A lists 41 farmed species spanning seven taxonomic orders, a list that may well grow in the future. Hymenoptera are the most numerous (10 species), mainly honey bees and stingless bees of the Apidae and, as of late, the giant hornet *Vespa mandarinia* (Smith 1852). Next come Coleoptera (9 species) such as *Tenebrio molitor* Linnaeus, 1758 (Tenebrionidae), *Alphitobius diaperinus* (Panzer, 1797) (Tenebrionidae), *Zophobas morio* (Fabricius, 1776) (Tenebrionidae), weevils of the genus *Rhynchophorus* (Curculionidae) and first rearing efforts with *Thysia wallichi* (Hope 1831); Lepidoptera (7 species), mainly silkworms of the genera *Bombyx* (Bombycidae) and *Antheraea* (Saturniidae), and Orthoptera (7 species) such as *Acheta domesticus* (Linnaeus, 1758) (Gryllidae), *Gryllodes sigillatus* (Walker, 1869) (Gryllidae), *Gryllus bimaculatus* (De Geer, 1773) (Gryllidae) and *Locusta migratoria* (Linnaeus, 1758) (Acrididae). To these are added Hemiptera exploited mainly for their secretions (3 species), Blattodea (3 species), and Diptera (2 species, notably *Hermetia illucens* (Linnaeus, 1758) (Stratiomyidae)).

**Figure 2:**
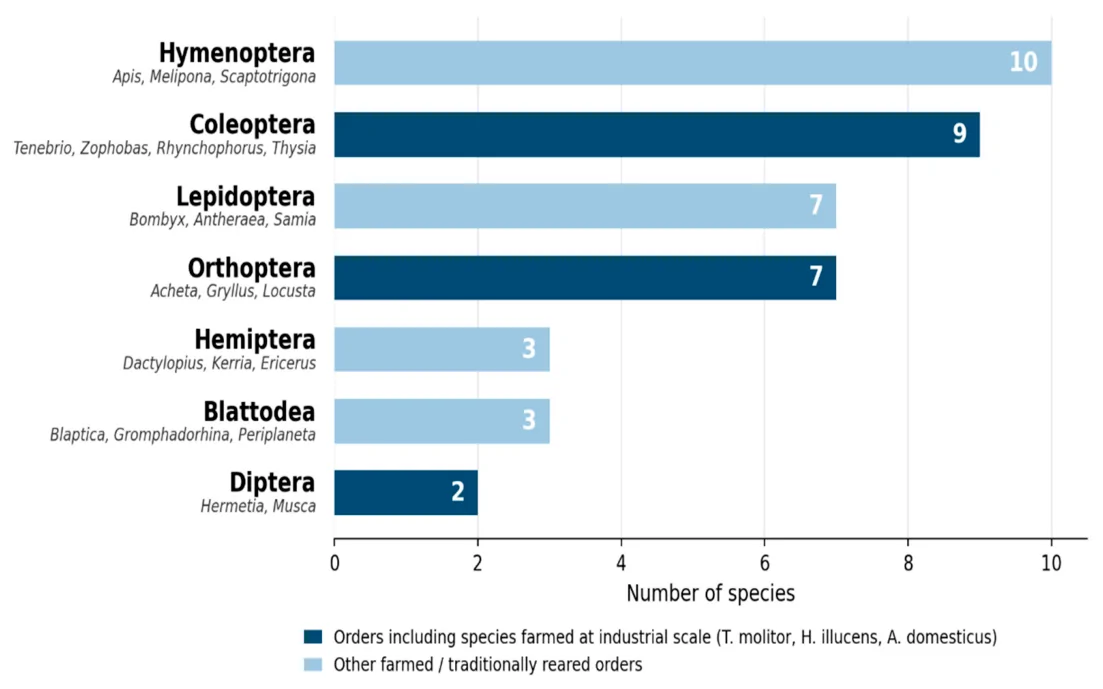
Distribution of farmed insect species by taxonomic order.

The intensity and purpose of rearing vary widely across species. To date, only a relatively limited range of species, notably *H. illucens* and *T. molitor*, together with *A. domesticus* and *Musca domestica*, is produced at large scale using relatively standardized rearing systems [44,48,49], whereas others belong to semi-industrial or artisanal value chains (sericulture, beekeeping, and meliponiculture, wasp rearing) or to cultures dedicated to derived products such as carmine or shellac. Depending on the relationship between insects, humans, plants, and the environment, rearing systems range from traditional practices close to the wild state to highly controlled systems in confined settings. Figure 2 shows the distribution of the 41 farmed species across the seven taxonomic orders identified in this review.

Among Orthoptera, grasshoppers and locusts (Acrididae) deserve to be considered as a food group in their own right, distinct from crickets. They are among the most widely consumed insects, notably in sub-Saharan Africa and Asia [50,51], yet only two species, *L. migratoria* and *Patanga succincta* (Johannson, 1763) (Acrididae), are regularly farmed (Appendix A). The reasons why grasshoppers could become a valuable food source for humans, including their nutrient profile, health risks, and consumer acceptance, have been reviewed in detail by Paul et al. [52], and Siddiqui et al. [51] provide a comprehensive overview of grasshoppers and locusts as human foods. Species with no farming tradition also show promise: the meadow grasshopper *Chorthippus parallelus* (Zetterstedt, 1821) (Acrididae) contains about 69% protein, with an excellent amino acid profile and a protein digestibility of 97%, and its commercial rearing has been judged feasible [53]. *L. migratoria* is produced industrially in the Netherlands and has been assessed as a safe novel food in the European Union [2,40], whereas *P. succincta* is reared in semi-industrial and artisanal systems in Southeast Asia [54]. Compared with crickets, acridids are more demanding to rear intensively: most species are fed on fresh grass or leaves rather than on formulated dry diets, locusts show density-dependent changes in physiology and disease resistance [55], and day length governs their growth and reproduction [56]. Most grasshoppers consumed today by humans are therefore still harvested from the wild [52], and data on how rearing conditions affect their nutritional and medicinal properties remain scarce, which makes this group a clear gap for future rearing research.

The farmed species listed in Appendix A include insects reared for different purposes, such as human food, animal feed, silk, honey and hive products, dyes, shellac, or traditional medicine and physiological research. Importantly, the fact that a species is primarily reared for animal feed or for other products does not exclude its use as human food. Indeed, the literature reports that certain species reared mainly for other purposes have developmental stages or products that are consumed by humans. For example, *H. illucens*, although primarily reared for animal feed, is included among the insect species reported as consumed by humans in the list compiled by Jongema [46]. Therefore, species reared for different purposes are considered in this review when the literature identifies them, or some of their developmental stages, as edible or consumed by humans.

Appendix A also indicates which species are represented in the 96-study core corpus: only 7 of the 41 species (*T. molitor*, *H. illucens*, *A. domesticus*, *G. bimaculatus*, *L. migratoria*, *B. mori*, and *A. mellifera*) contribute evidence to the main synthesis, whereas the remaining species belong to the taxonomic inventory only.

### 3.2. Rearing Systems for Edible Insects

Three main rearing systems can be distinguished: conventional, semi-intensive, and industrial (Table 1). They differ in their degree of control over the rearing environment, their level of technical sophistication and investment, and their impact on productivity and on the quality of the insects produced.

#### 3.2.1. Conventional (Extensive) Rearing Systems

Conventional systems correspond to traditional or artisanal rearing [60]. Several studies show that the rearing and consumption of edible insects remain concentrated in the tropical regions of Africa, Asia, and Latin America, where they provide both a traditional source of protein and income and where conventional systems continue to develop [2,61,62]. In Thailand, for example, many farmers rear bamboo caterpillars (*Omphisa fuscidentalis* (Hampson, 1896) (Lepidoptera: Crambidae)), stingless bees, lac-producing insects (*K. lacca*), or crickets for local food markets [63]. Such systems generally rely on rudimentary infrastructure built from local materials (wood, bamboo, dried leaves, or recycled containers) and feed insects on locally available organic resources such as agricultural residues, decomposing fruit, or fresh plant matter [61]. Their advantages include low set-up cost, accessibility for rural communities, and efficient use of local resources [2,17]; however, limited control of environmental parameters can produce considerable variability in nutritional quality and an elevated risk of microbiological and chemical contamination [64], a risk that also applies to wasp rearing in Japan [65] and Northeast India [66].

#### 3.2.2. Semi-Intensive Systems

Semi-intensive systems are an intermediate stage between traditional rearing and fully controlled industrial production [43]. They rest on a gradual shift toward tighter control, with some conditions managed while others still depend on biological or environmental factors. Insects are kept in simple installations (boxes, cages, pens, or small modules) that allow partial control of feeding, density, and, to a lesser extent, temperature and humidity [43]. These systems are especially well developed in parts of Asia, Africa, and Latin America, where the insects are fully domesticated and reared entirely in captivity [60]. Feeding is often standardized using enriched agri-food by-products or specific formulations that optimize growth and nutritional composition [28], while housing conditions are monitored and controlled [58]. The result is greater batch homogeneity, reduced sanitary losses, and improved biological performance, notably in feed conversion and protein yield [16]. Yields nonetheless remain variable because biological parameters are not fully controlled, and performance can fluctuate with substrate quality, population density, and local microclimate [28].

#### 3.2.3. Controlled Intensive (Industrial) Systems

Controlled intensive systems represent the most advanced level of insect farming, delivering standardized production in fully controlled environments [43], and are expanding rapidly in Europe, North America, and Asia [67]. They rely on specialized infrastructure that allows precise regulation of temperature, humidity, photoperiod, population density, and substrate composition. Insects are reared in optimized units (industrial farms, automated modules, or climate-controlled rooms) where the primary goal is to maximize productivity and feed conversion efficiency. Feed is formulated or standardized to ensure homogeneous, reproducible batch growth [2]. This approach also improves sanitary control, lowering the risks associated with pathogens and external contamination, and promotes high genetic stability and consistent nutritional and bioactive quality [43]. According to Halloran et al. [43], industrial rearing follows a large-scale production logic geared to the requirements of the food and feed markets. An overview of the industrialization history of edible insect rearing is provided by Megido et al. [68], and Pufpaff and McCann [69] deal with the rearing of *Periplaneta americana* (Linnaeus, 1758) (Blattodea: Blattidae), needed in China for health-related products.

### 3.3. Controlled Rearing Factors in Edible Insect Production

Edible-insect production depends strongly on rearing factors, which vary with the production system (Table 1). These factors (nutritional, environmental, zootechnical, and biological) directly affect the growth, survival, and nutritional quality of the insects.

#### 3.3.1. Nutritional Factors

Nutritional factors encompass the quality, composition, and availability of the feed provided. They are central to any rearing system because they directly govern growth, survival, reproduction, and nutritional composition [26,29,70]. The substrates used vary widely with the technical level of the operation. In traditional systems, insects are generally fed on local resources such as crop residues, leaves, decomposing fruit, or agricultural by-products; this poorly controlled feeding produces high variability in growth and nutritional quality [2,43]. In semi-intensive systems, farmers combine local resources with supplements (cereal brans, oilseed cakes, plant waste, or agri-food by-products) to improve nutrient availability and conversion efficiency [71]. Intensive or industrial systems, by contrast, use formulated, standardized diets balanced in protein, carbohydrate, lipids, vitamins, and minerals to meet each species’ requirements, thereby optimizing growth and feed conversion and enabling more homogeneous production and tighter quality control [72].

#### 3.3.2. Environmental Factors

Environmental factors mainly comprise temperature, relative humidity, photoperiod, and ventilation. These parameters strongly influence insect metabolism, growth, survival, and reproduction, and are controlled to differing degrees across systems and reared species [24,26,73,74,75,76,77]. In traditional systems, insects are reared under near-natural conditions with no regulation of environmental parameters; seasonal variation in temperature and humidity affects resource availability, developmental rate, and yields, producing high variability in growth and mortality [78]. Semi-intensive systems introduce partial control through shelters, cages, or simple structures that buffer extreme climatic swings, improving growth performance but remaining dependent on external conditions [43]. In industrial systems, environmental parameters are tightly controlled to achieve homogeneous, reproducible production [28], with temperature tuned to each species’ requirements; for example, in *A. domesticus*, *T. molitor*, and *H. illucens*, temperatures of 25–30 °C promote rapid growth, better feed conversion, and high biomass yield [74,79]. Relative humidity governs hydration, molting, and respiration. Too little causes dehydration, whereas excess promotes molds and pathogens [59]. In *A. domesticus*, optimal control improves survival and nymphal growth [80]. Ventilation complements this control by limiting the build-up of heat, humidity, and metabolic gases (CO_2_, ammonia), thereby improving rearing performance [13]. Finally, photoperiod regulates biological cycles, reproduction, and activity. In *L. migratoria*, day length influences growth, behavior, and egg-laying, allowing production to be optimized under controlled rearing [56].

#### 3.3.3. Biological Factors

Biological factors mainly concern the genetics of the reared populations, different sexes and developmental stages, and associated microbiota, which together shape growth, feed conversion, disease resistance, and biomass quality [81]. In traditional systems, insects are generally drawn from wild populations; the resulting lack of genetic control produces high variability in growth, fecundity, and survival, limiting production homogeneity [2]. In semi-intensive systems, empirical selection of the best-performing individuals can and should [82] gradually improve desirable traits of interest, although such practices remain poorly standardized [83,84]. In intensive rearing, genetic selection becomes a major optimization tool, improving growth rate, fertility, feed efficiency, and pathogen resistance [32,84]. In *B. mori*, domestication has yielded highly productive lines adapted to intensive sericulture [85], and comparable approaches are now emerging in edible insects to secure more stable, reproducible production. The gut microbiota are likewise essential, contributing to digestion, nutrient assimilation, and immunity. In *H. illucens* and *T. molitor*, microbiota drive substrate degradation and the production of metabolites that influence growth and feed valorization [86]. Controlling rearing conditions therefore helps stabilize the microbiota and improve the consistency of performance.

#### 3.3.4. Zootechnical Factors

Zootechnical factors (rearing density, feed management, developmental-stage sorting, and hygiene) influence growth, survival, feed conversion, batch homogeneity, and sanitary quality [87]. Density is critical: in traditional systems, poor control heightens competition, cannibalism, stress, and mortality, whereas semi-intensive and industrial systems adjust densities to developmental stage and species requirements. In *G. bimaculatus*, high densities reduce size uniformity and growth performance [88]. Feed management likewise affects biomass yield and conversion efficiency, with industrial systems relying on standardized diets for consistent growth [89]. Hygiene practices reduce microbiological risk. Traditional systems often lack effective waste management and disinfection, whereas intensive systems apply biosecurity measures (cleaning, substrate monitoring, and waste control) that limit production losses [59].

#### 3.3.5. Technological and Stress Factors

Rearing technology and stress management also shape production performance [90]. Traditional systems offer limited monitoring, whereas intensive systems use automated tools to regulate temperature, humidity, ventilation, and feeding, improving consistency [91]. Stressors such as high density, excessive handling, and environmental fluctuation impair growth, immunity, and survival; better control of zootechnical and environmental conditions mitigates these effects and enhances production stability [92,93].

## 4. Effects of Rearing Factors on the Qualities of Edible Insects

**Figure 3:**
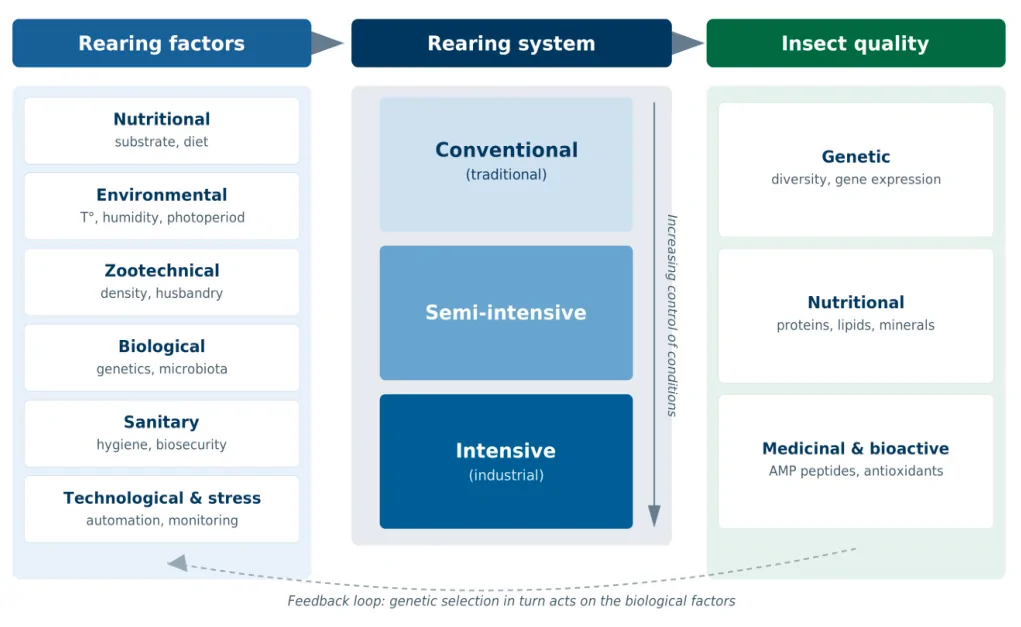
Conceptual framework linking rearing factors and systems to the genetic, nutritional, and medicinal properties of edible insects.

Rearing factors exert a decisive influence on the genetic, nutritional, and medicinal properties of insects, often differently in males and females [25] (Figure 3). Understanding these effects is essential for optimizing the quality of the insects produced for human and animal food. Controlled to increasing degrees according to the rearing system, these factors modulate insect properties in an interconnected manner, while genetic selection in turn acts on the biological factors, closing a feedback loop.

Before the action of each factor is examined, Table 2 provides an overall reading grid. For the three species reared most extensively at industrial scale (*T. molitor*, *H. illucens*, and *A. domesticus*), it cross-references each rearing factor (temperature, density, substrate, genotype, relative humidity, and photoperiod) with the measured effect on biochemical composition and performance. It thus consolidates the main quantitative values reported in the literature (otherwise scattered throughout the text) and serves as a guide for the following subsections, which set out the underlying mechanisms.

### 4.1. Influence on Genetic Properties

Rearing conditions act on insects at two distinct but complementary levels: at the epigenetic level, through reversible changes in gene expression that leave the DNA sequence intact; and at the genetic level *sensu stricto*, through changes in the structure and allele frequencies of populations.

#### 4.1.1. Epigenetic Effects and Phenotypic Plasticity

The phenotypic plasticity of insects gives them a remarkable capacity to adapt to variation in rearing conditions. Substrate composition, temperature, photoperiod, and population density can induce epigenetic modifications (DNA methylation, histone modifications, non-coding RNAs) that modulate the expression of genes governing growth, metabolism, and reproduction [30,31,94]. The most emblematic example of such nutritional control through the epigenetic pathway is the honey bee, in which reduced expression of *Dnmt3* directs larval development toward a queen or worker phenotype depending on diet [95]. These modifications directly shape observable traits: growth rate, survival, feed conversion efficiency, and biochemical composition [28,37]. In *H. illucens*, lipid content and fatty-acid profile vary markedly with the rearing substrate [96]. In conventional systems, the lack of control over rearing factors generates high phenotypic variability between batches and across seasons, a variability that is especially pronounced in insects, whose physiology is strongly governed by environmental conditions (notably temperature) owing to their ectothermy [94]. Industrial systems, by stabilizing abiotic conditions and standardizing diets, reduce this variability and yield insects with more reproducible phenotypes [43,97].

**Table 2:** Factors influencing the rearing and composition of three edible insect species (*T. molitor*, *H. illucens* and *A. domesticus*).

Species	Factor	Levels/Range Tested	Measured Effect (Values)	Source
*T. molitor*	Temperature	15, 20, and 25 °C	Nutritional composition shifts with temperature, with a moderate rise in nitrogenous compounds as temperature increases.	Adamkova et al. [98]
	Temperature	22 and 28 °C	Lipid content increases and crude protein decreases (from 64.33 to 54.41 g/100 g DM).	Kopecka et al. [99]
	Density	12 to 96 larvae/dm^2^	Higher density lowers individual weight gain, feed intake, and conversion efficiency.	Morales-Ramos & Rojas [100]
	Substrate	Organic by-products	Protein and lipid contents and conversion efficiency vary with substrate composition.	Van Broekhoven et al. [101]
	Genotype	Farmed populations/lines	Heritable variation in growth and composition, but low molecular diversity among farmed populations (inbreeding risk) and still-limited genomic resources.	Eriksson & Picard [32]
	Humidity	50–75% Humidity	Larval growth increases with relative humidity, which does not affect survival; the rearing optimum is generally around 60–75% RH.	Johnsen et al. [102]
	Photoperiod	Constant darkness (24D), 8L:16D, 16L:8D	Photoperiod affects developmental time and growth rate: survival and growth are highest and development shortest under constant darkness (a negatively phototactic species that prefers dark environments).	Eberle et al. [74]; Zim et al. [103]
*H. illucens*	Temperature	10 to 42 °C (nine constant temperatures)	Development rate increases with temperature between the lower developmental threshold (about 11–12 °C for larvae) and the thermal maximum (about 37–44 °C); population growth is optimal at 30 °C, with the highest intrinsic rate of increase and the shortest doubling time (5.5–5.7 days).	Chia et al. [79]
	Density	Larval density × nutrient concentration	Density and nutrient concentration modulate body protein and lipid contents.	Barragan-Fonseca et al. [70]
	Substrate	Biowaste and organic by-products	Larval protein content stays within a relatively narrow range, whereas crude lipid content depends strongly on dietary nutrient concentration and larval density.	Barragan-Fonseca et al. [70]
	Genotype	Selected lines and global populations	Selected vs. baseline: +39% larval weight, +34% wet crate yield, +26% dry-matter yield, +32% crude protein, and +21% crude fat per crate.	Facchini et al. [104]
	Humidity	25 to 70% humidity	Egg eclosion and adult emergence rise and developmental time falls with increasing RH; the optimum is generally >60% RH, and overly dry conditions cause larval desiccation.	Holmes et al. [105]
	Photoperiod	Light; 14L:10D; intensity and spectrum (UV–blue)	Unlike in larvae, light is essential to trigger adult mating and oviposition; mating probability increases with light intensity, and ~92% of matings occur during the photophase.	Zhang et al. [106]; Heussler et al. [107]
*A. domesticus*	Temperature	25 and 28 °C	At 25 °C: slower growth (0.51 mg/day DM), longer development (119 d), and higher adult mass (61 mg); at 28 °C: 0.99 mg/day, 49 d, 48 mg.	Booth & Kiddell [108]
	Density	0.09 to 0.93 individuals/cm^2^	Higher densities reduce survival to maturity and starvation resistance (stress, competition, cannibalism); optimal rearing is <0.93 individuals/cm^2^.	Mahavidanage et al. [109]
	Substrate	Plant by-products; varying dietary protein	Composition is substrate-sensitive: low-nutrient diets reduce protein content and increase lipid content.	Bawa et al. [110]
	Genotype	Farmed populations/lines (AdDV susceptibility)	Breeding programs are almost non-existent, genetic diversity is poorly characterized, and susceptibility to the densovirus (AdDV) is variable; *A. domesticus* is highly susceptible, driving replacement by more resistant species.	Szelei et al. [111]
	Humidity	65–75% humidity	Egg hatching is optimal at 30 °C and 65–75% RH; high RH benefits young instars but harms late instars and adults.	Mitchaothai et al. [80]
	Photoperiod	12L:12D; 14L:10D	A standard rearing parameter that entrains physiological rhythms (e.g., octopamine peaks after lights-off); direct effects on nymphal growth are poorly documented.	Woodring et al. [112]

Note: L = light (hours), D = dark (hours) per 24-h cycle (e.g., 16L:8D = 16 h light: 8 h dark); 24D = constant darkness.

#### 4.1.2. Effects on the Genetic Diversity of Populations

**Loss of genetic diversity in farmed insect populations**: In the absence of intentional selection, keeping insects in captivity is not genetically neutral. Genetic drift, founder effects, and inbreeding depression can gradually erode genetic diversity, reducing population robustness and adaptive capacity [34,35,113]. In the mealworm beetle *T. molitor*, for instance, a single cycle of sibling mating is enough to induce inbreeding depression, manifested as reduced adult mass and pre-imaginal survival and a lengthening of developmental time [114]. These effects are particularly pronounced in conventional systems that rely on small, isolated populations. Semi-intensive systems can partly offset them through periodic renewal of founders, but generally without a formal genetic-management program.

**Genetic selection and improvement of farmed insect populations:** In intensive systems, genetic selection can steer populations toward traits of economic interest, an approach first suggested by Meyer-Rochow [82]. Recent work shows that growth rate, fertility, pathogen resistance, and biochemical composition are heritable enough to respond to selection [32]. In *H. illucens*, estimates from genome-wide single-nucleotide polymorphism (SNP) data confirm additive genetic variation that can be exploited for growth traits (narrow-sense heritability of about 0.2 for pupal weight) [115]. Applied-genomics tools (high-throughput sequencing, SNP markers, and genomic selection) open new avenues for accelerating genetic improvement beyond classical phenotypic approaches [84]. The case of *B. mori*, whose millennia of domestication have produced more than 4000 selected lines yielding up to ten times more silk than the wild species *Bombyx mandarina* (Moore, 1872) (Bombycidae), illustrates the long-term potential of this genetic lever in farmed insects [116].

### 4.2. Influence on Nutritional Properties

The nutritional composition of edible insects is not a fixed, intrinsic characteristic. It varies considerably with rearing conditions, and the developmental stages of an insect and special diets have to be identified for different insects under cultivation [26]. This makes it a direct lever for optimizing the nutritional quality of the insects produced.

#### 4.2.1. Effect of the Feed Substrate

The nature and composition of the substrate are the most extensively documented factor in the edible insect rearing literature [16,36,117,118]. Diet acts through a relatively direct transfer between ingested nutrients and those accumulated in larval tissues [119].

Substrates enriched in unsaturated lipids (linseed, hemp, or rapeseed oils, omega-3-rich by-products) are directly reflected in the larval fatty-acid profile, raising the polyunsaturated fraction of nutritional interest [37,120,121]. This lever has already been exploited deliberately in *H. illucens*. Prepupae reared on diets containing fish offal stored about 3% of their lipids as omega-3 fatty acids, including eicosapentaenoic (EPA) and docosahexaenoic (DHA) acids, whereas those reared on cow manure alone contained negligible amounts; a comparable enrichment was obtained when the offal was supplied only during the final day before prepupation, showing that the timing of supplementation matters as much as its level [122]. Seaweed-enriched media likewise raised the omega-3 content of the larvae [123], and supplementing the substrate with oilseeds shifted larval fat toward a more unsaturated profile [120]. A recent review concluded that the larval fatty-acid profile closely tracks that of the substrate, so that fatty acids with more than 18 carbons must be supplied in the diet to be found in the larvae [124]. The scope for enrichment nonetheless has limits: lauric acid (C12:0), synthesized de novo from dietary carbohydrates, usually dominates the larval fat, so that the polyunsaturated fraction obtained depends on the fat source, its inclusion level, and the carbohydrate content of the substrate [37,120,124]. Conversely, substrates poor in lipids or dominated by carbohydrates channel larval metabolism toward de novo lipogenesis, yielding predominantly saturated fatty acids [37].

In *T. molitor*, the response is structured by the balance among macronutrients, in line with the nutritional geometry framework [125]. Larvae actively regulate intake toward a target of protein and carbohydrate. Protein-rich diets improve growth and biomass gain and shorten developmental time, whereas excess lipid or an overly energetic substrate favors fattening at the expense of the protein fraction [36,118]. The origin of dietary nitrogen further influences not only the quantity of protein accumulated but also its amino acid profile, a decisive parameter for final nutritional value [98,101].

This substrate sensitivity offers a twofold applied benefit. It enables the bioconversion of agro-industrial by-products into high-value biomass, while demanding standardization, since the compositional variability of substrates carries directly through to the consistency of the biomass produced. Substrate formulation is thus at once the principal lever for nutritional optimization and the principal source of variability to be controlled in scale production. To implement this knowledge, it is essential to possess information on the species of plants that are acceptable to the insect. Islam et al. [126] reported that different varieties of mulberries could affect key chemical components of silkworms, which is why Mitsuhashi and Sato [127] provided a list of 22 plants that were acceptable to *Rhynchophorus ferrugineus* (Olivier, 1790) (Coleoptera: Curculionidae).

#### 4.2.2. Interaction Between Genotype and Nutritional Composition

The nutritional composition of farmed insects does not depend on the feed substrate alone. It is also shaped by the genotype and, above all, by the interaction between the two. In *H. illucens*, genetically distinct strains differ in lipid and protein contents and in amino-acid profile, and show strong genotype × diet interactions, revealing that lines adapt differently to the same substrate [128]. A comparison of two genetically distinct *H. illucens* populations showed that genotype modulates lipid composition independently of diet [129]. This variability rests on a substantial heritability of body-composition traits: heritable variation in body size and composition, demonstrated with a related-family design at the two main larval stages, indicates real scope for improvement by selection toward nutritional profiles of interest, complementing substrate control [130]. This potential is vividly illustrated by an industrial selection program launched in 2019, which achieved a 39% increase in *H. illucens* larval weight after only 16 generations of selection for greater body mass [104], showing how rapidly substantial genetic gains can be obtained under industrial rearing. Together, these convergent results indicate that nutritional optimization cannot rely on substrate control alone: identifying and selecting lines with stable, favorable composition profiles is a complementary lever, particularly relevant for value chains aiming at nutritional standardization for food or nutraceutical purposes.

#### 4.2.3. Effect of Temperature

Rearing temperature is among the most decisive abiotic factors for the metabolism of ectothermic insects, whose biochemical composition responds directly to ambient thermal conditions [27]. The effect of temperature on the protein and lipid fractions is the best documented aspect. In *T. molitor*, larval body weight, dry-matter content, and fat concentration increase with rearing temperature (fat rising from 22.46 to 36.01 g/100 g Dry Matter between 22 and 28 °C), whereas crude protein falls significantly, from 64.33 to 54.41 g/100 g Dry Matter across the same range. This inverse relationship between lipid accumulation and protein content reflects a preferential activation of lipogenic pathways at high temperature, at the expense of protein synthesis [99]. Bjorge et al. [27] confirm this trend, reporting that nutritional composition in *T. molitor* varies with rearing temperature. Larvae reared at 31 °C show the highest lipid content (47.4% of dry matter), whereas lower or higher temperatures reduce lipids and raise proteins, pointing to an optimal thermal range for the species. Beyond macronutrients, temperature affects protein quality. Kopecka et al. [99] show that methionine is the limiting amino acid in *T. molitor* and that the essential amino acid index (EAAI) varies between 37 and 45% with rearing temperature, indicating that qualitative (not merely quantitative) protein value is temperature-dependent. Adamkova et al. [98] note that temperature- and diet-related changes are most pronounced for the essential amino acids valine, arginine, and leucine, suggesting that the metabolic pathways governing their synthesis or catabolism respond differently to temperature.

Temperature also shapes lipid quality through homeoviscous adaptation. As ectotherms, insects adjust the unsaturation of their membrane fatty acids to maintain membrane fluidity under prevailing thermal conditions. This entails a higher unsaturated/saturated fatty-acid ratio at low temperatures, where unsaturated fatty acids preserve fluidity and protect the function of enzymes and ion channels. Larvae reared in a cool environment therefore tend to show a higher proportion of polyunsaturated fatty acids (PUFAs), favorable to nutritional quality for human and animal food, whereas high temperatures shift synthesis toward saturated fatty acids via de novo lipogenesis [98]. Crucially, the effect of temperature is not independent of the feed substrate: the biochemical response to temperature varies with diet composition, and vice versa [98,121]. In a standardized production logic, the two parameters must therefore be controlled jointly. This interaction also opens a useful applied prospect. A *T. molitor* meal of controlled nutritional value can be obtained (protein 546 to 657 g/kg, lipids 166 to 295 g/kg and total amino acids 415 to 475 g/kg) through a temperature–diet regime controlled between 15 and 25 °C, concretely demonstrating the scope for steering nutritional quality by managing rearing conditions [98].

#### 4.2.4. Effect of Density and Sanitary Conditions

Excessive rearing density generates intraspecific competition for feed that lowers individual nutrient intake, reduces weight gain, and degrades biochemical quality [70,101]. Microbial contamination, favored by poor hygiene, can further degrade nutritional quality by altering insect proteins and lipids [131].

#### 4.2.5. Effect of Developmental Stage at Harvest and Sex

Beyond environmental factors, biological factors tied to husbandry decisions, namely the developmental stage at which insects are harvested and the sex of the individuals harvested, also modulate nutritional composition. Pupae of *B. mori*, a by-product of sericulture, illustrate this point. As shown by Kotake-Nara et al. [132], their oil is rich in polyunsaturated fatty acids, notably α-linolenic acid, which gives it nutritional value but also makes its oxidative stability a quality criterion to be controlled. In their review of the nutritional value of silkworm pupae, Hăbeanu et al. [133] confirm a fatty-acid profile of interest for human and animal food, while stressing that composition varies with strain, diet, and processing. Using lipidomic profiling, Hu et al. [134] further revealed marked differences in lipid composition between male and female pupae, showing that sex is a source of compositional variability in its own right. Likewise, Hou et al. [135] showed that the hemolymph proteome of *B. mori* changes substantially across developmental stages, confirming that the stage chosen for harvest shapes the protein profile of the product. Harvest stage and, where feasible, sex sorting are therefore additional levers for standardizing the nutritional quality of farmed insects, alongside substrate and temperature control.

### 4.3. Influence on Medicinal and Bioactive Properties

Beyond their nutritional value, edible insects contain a range of compounds of growing medical and pharmaceutical interest: antimicrobial peptides, antioxidants, anti-inflammatory and antihypertensive molecules, and polysaccharides, polyphenols, and bioactive fatty acids [8,10,136]. Medicinal insects have also been reported in connection with malaria, hypertension, burning sensation, virility, strength, and use as aphrodisiacs [11,137]. The nature and concentration of these compounds are not fixed; they are strongly conditioned by rearing conditions.

In conventional systems, insects feed on a wide variety of substrates (medicinal plants, mixed vegetation, other insects, etc.), which favors the accumulation of broad-spectrum bioactive compounds. It is precisely this richness in predominantly plant-derived molecules that underlies the therapeutic use of wild-collected species, as documented in the ethnozoological practices of sub-Saharan Africa and Asia [50,138,139,140]. Industrial systems follow the opposite logic: by standardizing substrates, they make it possible to target and reproduce precise bioactive profiles rather than relying on natural dietary diversity, opening the way to calibrated functional ingredients for nutraceutical or pharmaceutical use [141].

#### 4.3.1. Antioxidant Compounds

The antioxidant activity of edible insects is closely tied to the antioxidant richness of their feed substrate [142]. Insects reared on substrates rich in plant polyphenols (aromatic herbs, soybean meal, plant extracts) show higher antioxidant activity than those fed phytochemical-poor diets [143,144]. In *A. domesticus*, an organic diet enriched with alfalfa, soybean, and maize was associated with a marked rise in phenolic content and with in vitro antioxidant activity exceeding that of the feed extracts themselves, the insect apparently able to absorb and concentrate diet-derived phenolics [144]. These properties are attributed to the accumulation of diet-derived polyphenols and to the synthesis of antioxidant peptides during the digestion of plant proteins. Antioxidant activities have also been reported from silkworm pupae and many other edible insect species, in which substances such as bioactive peptides, phenolic compounds, vitamins, carotenoids, and trace minerals enhance the cellular redox homeostasis and protect cells against oxygen radicals [145].

#### 4.3.2. Bioactive Peptides from Protein Digestion

Edible insects are a source of bioactive peptides released during gastrointestinal protein digestion. These peptides display antihypertensive (angiotensin-converting enzyme inhibition), antioxidant, anti-inflammatory, antimicrobial, and antidiabetic (DPP-IV and α-glucosidase inhibition) activities [146,147]. In *T. molitor*, peptides obtained by enzymatic hydrolysis inhibit the lipoxygenase (LOX) and cyclooxygenase (COX) enzymes involved in inflammation [148]. The composition and concentration of these peptides depend directly on the protein content of the feed substrate and on post-harvest processing.

#### 4.3.3. Endogenous Antimicrobial Peptides (Innate Immunity)

As part of their innate immunity, insects naturally produce antimicrobial peptides such as defensins, cecropins, attacins, and melittin [149]. Their production is stimulated by immune stress and by rearing conditions [92,150]. High densities, which raise pathogen pressure, can induce overproduction, whereas strict sanitary conditions reduce immune stimulation and may lower antimicrobial peptide concentrations [55]. This tension between sanitary optimization and the production of bioactive compounds is a challenge specific to insect farming oriented toward medicinal properties.

#### 4.3.4. Thermal Modulation of Bioactive Biosynthesis Pathways

Rearing temperature is a well-established determinant of the primary nutritional composition of insects. In *T. molitor*, protein and lipid contents, as well as certain amino acid profiles, vary significantly with temperature [98]. Beyond nutritional traits, thermal stress also alters cellular responses, inducing oxidative stress, modulating antioxidant enzyme activities (SOD, CAT), and promoting overexpression of the heat-shock proteins HSP70 and HSP90, providing evidence of metabolic reprogramming in response to temperature [151]. These physiological adaptations do not, however, demonstrate that temperature directly regulates the accumulation or biosynthesis of secondary bioactive compounds (phenolics, flavonoids, terpenoids, etc.); to date, no study has established such a relationship in edible insects. Adamkova et al. [98] likewise stress that the effects of temperature, alone or combined with diet, on these compounds remain to be elucidated. The available evidence concerns mainly dietary factors, as shown by the incorporation of carob pods (*Ceratonia siliqua*), which raises phenolic content and antioxidant activity in *T. molitor* larvae [142].

**Figure 4:**
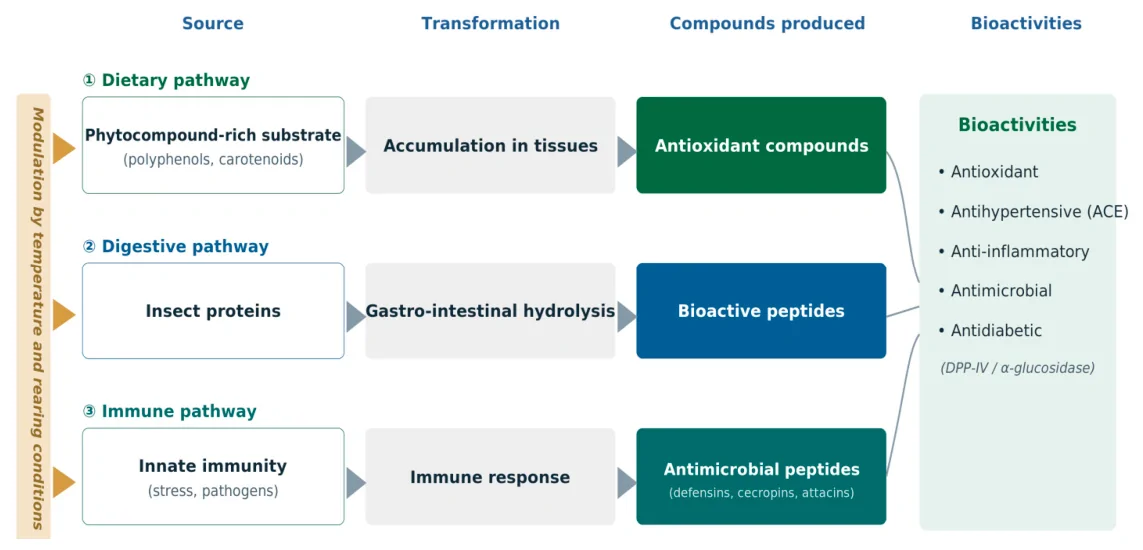
Origin of the bioactive compounds of edible insects.

The effect of temperature on the regulation and accumulation of bioactive compounds therefore remains a biologically plausible hypothesis awaiting experimental validation. Overall, insect bioactivity arises from the interaction of several biological pathways (Figure 4): a dietary pathway, through the accumulation of antioxidant phytocompounds; a digestive pathway, through the release of bioactive peptides during protein hydrolysis; and an immune pathway, through antimicrobial peptides of innate immunity. Temperature and rearing conditions may modulate the intensity of these pathways, although their specific contribution to bioactive profiles remains insufficiently characterized.

### 4.4. Comparative Synthesis: Convergences, Divergences, and General Trends

Bringing the reviewed studies together reveals several convergent trends. First, the three dimensions examined (genetic, nutritional, and medicinal) respond to the same rearing levers, foremost the feed substrate and temperature, the two most thoroughly documented and reproducible factors in the literature. Second, insect composition emerges unanimously as a plastic rather than an intrinsic trait. Whether for the protein and lipid fractions, antioxidant activity, or the essential amino-acid index [99], studies agree that it can be steered by rearing conditions. Third, a consensus emerges on the potential for genetic improvement. The substantial heritability of composition traits [130] and the rapid gains achieved through selection (+39% larval weight over 16 generations in *H. illucens* [104]) together identify the genotype as a complementary and still under-exploited lever.

Divergences nonetheless persist. The relative sensitivity of the biochemical fractions to the substrate differs among species. In *H. illucens*, the lipid fraction is far more responsive than the stable protein fraction, whereas in *T. molitor* it is the balance among macronutrients, interpreted through nutritional geometry, that structures the response [70,125]. This heterogeneity stems partly from disparate protocols (substrates, temperatures, developmental stages, and rearing durations) that are rarely comparable across studies, limiting quantitative pooling. The divergence is especially clear for the protein response to temperature. In *T. molitor*, Kopecka et al. [99] report that raising the temperature from 22 to 28 °C increases lipid content and decreases crude protein, whereas Adamkova et al. [98] observe a moderate rise in nitrogenous compounds with temperature, while stressing that substrate influences this parameter far more decisively than temperature itself. The very direction of the thermal effect on the protein fraction is thus not settled and remains conditioned by diet and protocol.

Several confounders may explain this discrepancy. The two studies explored different thermal windows (22–28 °C versus 15–25 °C), used different substrates, and did not necessarily harvest larvae at the same developmental stage or after the same exposure duration. In our view, developmental stage at harvest, which is closely coupled with exposure duration, is the most likely explanation. Because development accelerates with temperature, larvae reared at a higher temperature and harvested at a fixed age are physiologically older and have accumulated more fat-body lipid, which dilutes the protein fraction; the nutrient composition of *T. molitor* larvae is indeed known to change markedly with age and stage [77]. Diet composition is the second most likely confounder, since Adamkova et al. [98] themselves found substrate to influence nitrogenous compounds more strongly than temperature, and the thermal response varies with diet [98,121]. The non-linear response reported by Bjorge et al. [27], with a lipid maximum at 31 °C, further indicates that results obtained over different temperature ranges cannot be compared directly. Moreover, male and female individuals, whose lipid profiles already differ, as shown by Hu et al. [134], may not respond in the same way. Resolving this question therefore requires dedicated experimental designs, which we identify as an explicit research priority (Section 5.4.5). A strong taxonomic imbalance compounds the problem: most data come from three industrial species (*T. molitor*, *H. illucens*, *A. domesticus*), while the medicinal dimension is the least standardized and the most dependent on substrate variability.

Beyond convergences and divergences, comparing the three dimensions reveals interactions and sometimes tensions among optimization objectives. The genotype × substrate interaction shows that nutritional optimization cannot rely on dietary control alone [128,129], while thermal modulation acts simultaneously on primary composition and on the biosynthetic pathways of bioactive compounds. Above all, a tension emerges between standardization and functional richness. The controlled industrial conditions that ensure nutritional reproducibility tend to impoverish the substrate diversity underlying bioactive compound accumulation, which conventional systems favor instead. This trade-off is one of the most decisive points of rearing management and directly shapes research priorities.

These elements highlight the main gaps in the literature: the insufficient standardization of rearing protocols, the under-representation of non-industrial species and of the medicinal dimension, and the still under-exploited genetic lever.

## 5. General Discussion, Limitations, and Perspectives of Insect Farming

### 5.1. Critical Synthesis of Rearing Systems

#### 5.1.1. Comparison of Conventional, Semi-Intensive, and Industrial Systems

**Figure 5:**
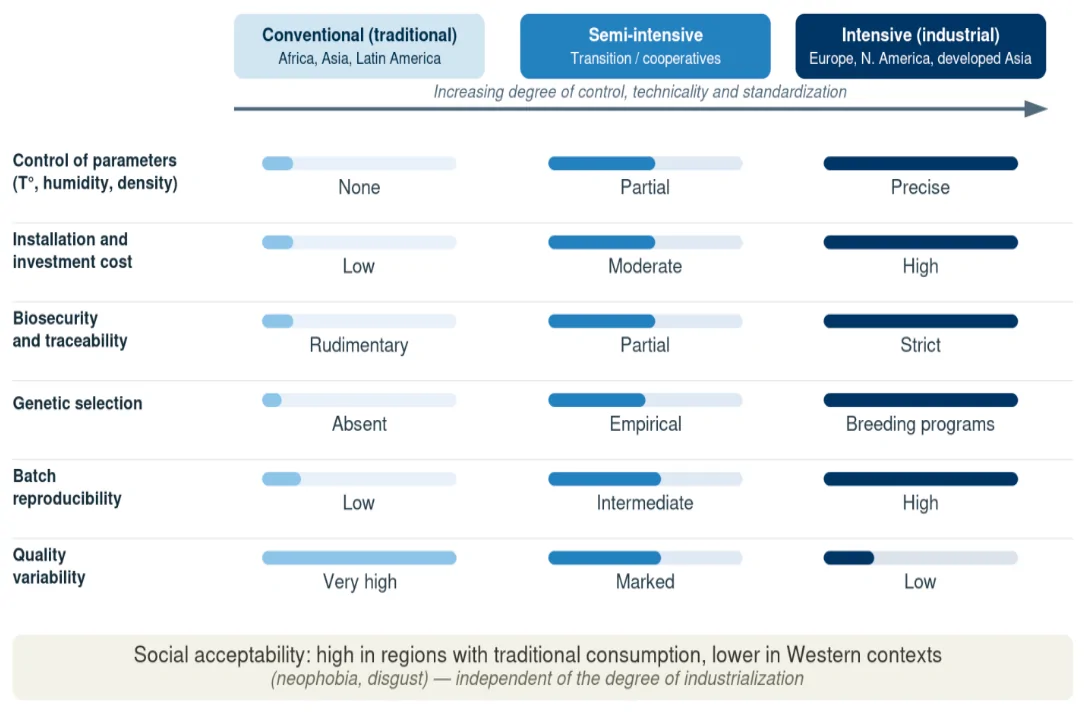
Continuum of edible insect rearing systems.

The comparative analysis of the three main rearing systems reveals a continuum (Figure 5) of control over production conditions, with direct implications for the quality and consistency of the insects produced [14,152]. At one end, traditional systems offer low set-up costs and integrate naturally into the food and economic practices of rural communities, particularly in sub-Saharan Africa, Asia, and Latin America [2,66], and offer benefits to local enterprises [153]. They valorize local resources efficiently and maintain a direct link with regional entomological biodiversity. However, limited control of environmental, nutritional, and sanitary parameters generates substantial variability in quality and yields, constraining their contribution to stable, standardized food production [64].

#### 5.1.2. Technical and Economic Limitations

Several technical obstacles hinder the large-scale development of insect farming. Mechanization and automation of the rearing steps (feeding, stage sorting, harvesting, post-harvest processing) remain incomplete, sustaining a strong dependence on manual labor that raises production costs [154,155]. The cost of insect protein still exceeds that of conventional sources such as soybean or fishmeal, limiting short-term competitiveness, particularly in animal nutrition [156]. Moreover, variability in species, strains, and practices across farms complicates the standardization of processes and finished products, hampering industrialization [157]. Finally, regulatory constraints (notably those on the substrates authorized for each species) restrict the valorization of organic waste and limit the expected circularity gains [158].

#### 5.1.3. Constraints Related to the Production of Edible Insects

Despite its strong potential, insect farming faces four major constraints. The first is the variability of biochemical composition (proteins, lipids, fatty acids, micronutrients), arising from the interaction of biological, nutritional, environmental, and zootechnical factors; particularly marked in conventional and semi-intensive systems, it is aggravated by the absence of harmonized analytical protocols and complicates compliance with market requirements [37,97,128].

The second constraint is genetic. Selection programs remain underdeveloped, closed populations are exposed to inbreeding and diversity erosion, genotype–environment interactions are poorly characterized, and reference genomic resources are lacking for most species except *B. mori* and, more recently, *H. illucens*, *T. molitor*, and *Protaetia brevitarsis* (Lewis, 1879) (Coleoptera: Scarabaeidae) [32,84,159].

The third constraint is sanitary. Reared at high density on varied substrates, insects can be contaminated by bacteria (enterobacteria, spore-formers, more rarely Salmonella or Listeria) and molds [160], and heavy metals bioaccumulate, unlike mycotoxins and pesticides, which are generally excreted; without harmonized regulatory thresholds, only industrial systems (HACCP, traceability) provide solid guarantees [161,162]. Heavy-metal accumulation is, moreover, species- and element-specific. When substrates were spiked with cadmium, lead, and arsenic at 0.5 to 2 times the EU maximum levels for complete feed, *H. illucens* larvae accumulated cadmium strongly (bioaccumulation factor above 6) and lead slightly (about 1.2–1.4), whereas *T. molitor* larvae accumulated neither cadmium (factor below 1) nor lead (about 0.05) but did accumulate arsenic (1.4–2.6) [163]. Larval concentrations can therefore exceed regulatory limits even when the substrate complies with them, which led the authors to call for species-specific maximum levels [163]. Direct comparisons of contaminant levels across rearing systems are still lacking; exposure is nevertheless likely to be highest in conventional systems, where insects feed on uncontrolled organic residues or are collected in polluted environments, and lowest in industrial systems restricted to feed-grade substrates, as currently required under EU legislation [158,161]. Substrate-side control is therefore the main mitigation lever: sourcing substrates of known origin, screening them for cadmium, lead and arsenic before use, excluding high-risk side streams for strongly accumulating species, and matching each species to substrates according to its element-specific accumulation profile [161,163]. The fourth is social acceptability. In the West, neophobia and disgust limit consumption (13 to 15% of consumers in Europe), even though processed forms such as flours and clever advertising may improve it [164,165]. Acceptance remains high in some countries where entomophagy is culturally rooted [166,167], although not in all of them, as Muller [168] has shown for Laos and Thailand. Sustainable development of the sector thus requires concerted efforts in production standardization, genetic improvement, sanitary control, and consumer awareness [169].

Table 3 summarizes, for each of the three rearing systems, the main strengths and weaknesses relating to these four constraints, and highlights the recurring trade-off between the low cost and high acceptability of traditional systems and the quality control and biosecurity offered by intensive systems.

### 5.2. Development Opportunities

#### 5.2.1. Global Food Security

Insect farming offers a credible response to global food-security challenges in the face of the projected world population growth (despite declines in European and East Asian countries). With a feed conversion ratio two to five times better than that of conventional animal proteins, production feasible on very small land areas and with far lower water use, and a markedly smaller carbon footprint, insects are a high-potential alternative protein source [14,20]. In developing countries, where entomophagy is often already embedded in food traditions but still relies largely on seasonal wild harvesting, developing controlled farming would help regularize and expand supply, improving nutritional security for vulnerable populations and augmenting living standards [2,5]. Realizing this potential depends closely on the rearing system: conventional and semi-intensive systems, accessible and inexpensive, suit local food security in developing countries despite variable quality, whereas industrial systems provide the stable, standardized, and sanitarily controlled production required for mass supply.

#### 5.2.2. Circular Economy and Waste Valorization

**Figure 6:**
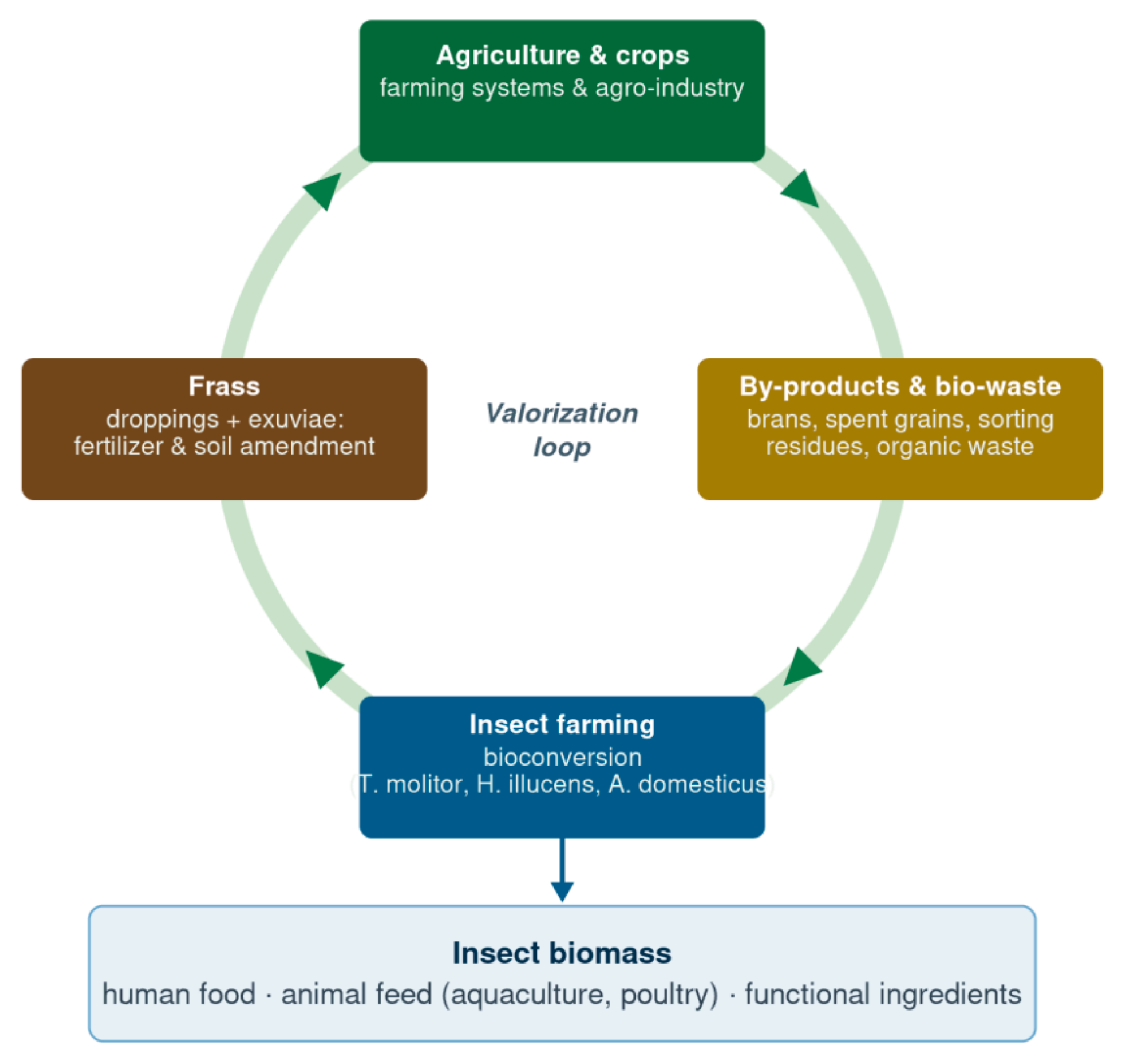
Circular economy of insect farming.

One of the most promising opportunities of insect farming is its capacity to integrate into circular-economy models (Figure 6). Insects, particularly *H. illucens* and *T. molitor* (to some extent, gryllid species as well), can convert large volumes of organic waste (agricultural residues, agri-food by-products, fermentable household waste) into high-value protein biomass and into frass usable as a biofertilizer [170]. *H. illucens* can reduce organic biomass by 50 to 60% while producing larvae rich in protein and lipids for animal and human nutrition [171]. This dual function (waste bioconversion and nutrient production) gives insect farming a strategic role in the transition toward circular, resilient food systems [152]. The frass produced also offers valuable agronomic properties as an organic amendment, closing the nutrient cycle [172] and, in some cases, can and has been used medicinally [8]. This valorization nonetheless depends on the rearing system. The nature and quality of the substrate, which determine the extent of bioconversion, simultaneously govern the sanitary safety of the product (bioaccumulation of heavy metals and microbial load), so that the circular pathway is fully compatible with human food use only in systems able to control and trace their inputs.

#### 5.2.3. Sustainable and Functional Nutrition

Beyond their crude protein value, edible insects offer still largely untapped prospects for nutritional and nutraceutical valorization. Their richness in bioactive peptides, essential fatty acids, bioavailable minerals, and prebiotic chitin opens avenues for functional foods targeting the prevention of chronic disease, including cardiovascular disease, type 2 diabetes, and obesity [173,174]. The ability to modulate insect biochemical composition through rearing conditions (notably enrichment in omega-3 fatty acids by manipulating the substrate, as already demonstrated in *H. illucens* larvae [122,123,124]; Section 4.2.1) provides a lever for developing differentiated, high-value products [123].

### 5.3. Operational Recommendations for Optimizing Rearing Practices

Beyond the research directions discussed below, this review yields practical recommendations for improving insect quality for consumption. These recommendations apply across the three rearing systems and should be tailored to the species, available technical capacity, and target market (food, nutraceuticals, or animal feed).

**Substrate formulation**: Select and, where possible, standardize the substrate according to the target profile: favor substrates rich in unsaturated lipids and plant polyphenols to maximize the polyunsaturated fraction and antioxidant activity, or more protein-rich substrates to boost protein content. Controlling the sanitary quality of inputs (heavy metals, microbial load) is a prerequisite wherever human food use is intended [36,37,121,142,163].

Thermal control: Keep temperature within the optimal range for the species (generally 25–30 °C for *T. molitor*, *H. illucens*, and *A. domesticus*); lower it when the aim is to enrich the biomass in polyunsaturated fatty acids of nutritional interest, accepting the trade-off with growth rate. Because their effects interact, steer temperature and substrate jointly rather than in isolation [27,74,98].

**Density and biosecurity**: Adjust rearing density by stage to limit feed competition, cannibalism and stress, which degrade weight gain and biochemical quality; apply hygiene and biosecurity protocols (cleaning, microbiological control of substrates, frass and waste management, HACCP principles in industrial systems) to secure the sanitary quality of batches [59,70,88,162].

**Genetic management of populations**: Prevent the erosion of genetic diversity and inbreeding through periodic renewal of breeders and maintenance of sufficient founder populations; where possible, favor lines with stable composition to secure reproducibility beyond dietary control alone [128,130].

**Standardization and traceability**: Document and harmonize rearing parameters (substrates, temperatures, stages, durations, harvesting, and post-harvest processing) within the farm to ensure batch consistency and ease compliance with food-market requirements. Explicitly arbitrate between nutritional standardization (food sectors) and richness in bioactive compounds (nutraceutical or medicinal applications) according to the production goal [97,141,157].

### 5.4. Research Perspectives

#### 5.4.1. Genetic Improvement, Substrate Optimization, and Standardization

Structured genetic selection is the most urgent priority. Applying genomic tools (whole-genome sequencing, SNP markers, GWAS, genomic selection) to priority species and building genetic-resource collections would accelerate the improvement of key traits while limiting inbreeding [123,175]. In parallel, the use of sustainable, locally available substrates (agri-food by-products and organic side-streams) and the harmonization of rearing protocols and analytical methods across laboratories are essential to improve nutritional quality and the comparability of data [176].

#### 5.4.2. Standardization of Systems and Analytical Methods

The absence of standardized rearing protocols and of analytical methods harmonized across laboratories is one of the main barriers to comparing the available data [157,176]. Developing international guidelines for experimental rearing (abiotic conditions, substrate compositions, harvesting methods, analytical protocols) is essential to enable rigorous inter-study comparison and to identify transferable best practices. Such standardization also underpins robust regulatory frameworks for approving insects as novel foods and food ingredients internationally.

#### 5.4.3. Sustainable Industrialization and a Systemic Approach

The industrialization of insect farming raises sustainability challenges that extend beyond yield optimization. A systemic approach (integrating life-cycle analysis of production systems, assessment of substrate environmental impact, management of the welfare of reared insects, and the socio-economic constraints of local actors) is needed to steer the sector toward genuine sustainability [177,178]. Digital monitoring technologies (connected sensors, artificial intelligence, digital twins of farms) offer promising means of optimizing rearing conditions in real time and reducing losses [179,180]. Finally, training field actors (farmers, technicians, processors) and transferring knowledge between research centers and industry are indispensable to the success of this transition toward a sustainable, inclusive, and scientifically grounded insect farming [181,182].

#### 5.4.4. Temperature and Secondary Metabolism: A Still Unexplored Axis

The specific effect of rearing temperature on the biosynthesis of secondary bioactive compounds (alkaloids, flavonoids, terpenoids, phenolics) remains largely unexplored in farmed edible insects, and so does a possible effect on the presence of antinutrients, which have been reported from a number of edible species, e.g., ants, termites, crickets, and grasshoppers, to name but a few ([26] and Oibiokpa et al. [183]). While the link between temperature and primary nutritional composition (proteins, lipids, amino acids) is well established, its extension to secondary metabolic pathways remains a mechanistically plausible but experimentally unverified hypothesis, and thus a priority research axis for species such as *T. molitor* and *H. illucens* [98].

#### 5.4.5. Disentangling the Effect of Temperature on Protein Content

The opposite directions reported for the effect of rearing temperature on crude protein in *T. molitor* [98,99] (Section 4.4) should be treated as an explicit research priority rather than as residual heterogeneity, because protein content is a key quality criterion for insect-based foods and the uncertainty directly affects how temperature should be set in production. We recommend factorial experiments crossing at least three temperatures spanning the 20–31 °C range with contrasting diets (for example, protein-rich versus carbohydrate-rich substrates), with larvae harvested both at a fixed developmental stage and at a fixed age, so that the effects of temperature, diet, developmental stage, and exposure duration can be separated. Reporting composition on a dry-matter basis, together with larval age, instar, and rearing duration, would make future results directly comparable across studies.

### 5.5. Limitations of the Study

#### 5.5.1. Critical Nature of the Review and Absence of Formal Risk-of-Bias Assessment

As a methodological choice, this work is a critical (narrative) rather than a systematic review. Study selection followed a structured, explicit approach (databases searched, inclusion and exclusion criteria, multi-step screening), but without a preregistered protocol or exhaustive documentation of the search strings for each database. Suited to the emerging, cross-cutting nature of the field, this framework favors integrative reading over procedural exhaustiveness. Consistently, the included studies were not subjected to a standardized assessment of methodological quality or risk of bias, and the synthesis remained qualitative and comparative, without meta-analysis. Because selection relied on a limited number of reviewers, selection bias cannot be entirely ruled out; the conclusions should therefore be read as synthesis trends rather than quantified estimates.

Several sources of bias may also affect the primary studies underpinning this synthesis. Many rely on small samples, single batches, or a single production facility, with limited or no replication, which increases the risk of chance findings and constrains external validity. Rearing factors are often correlated in practice (temperature, for instance, co-varies with development time and substrate moisture), which is why reported effects may be partly confounded rather than attributable to a single variable. Because the included studies were not appraised with a formal instrument (such as SYRCLE- or ROBINS-type tools for experimental and observational designs), differences in randomization, blinding, environmental control, and completeness of outcome reporting could not be weighted systematically. A reporting bias toward statistically significant or favorable results is also plausible in an applied, commercially driven field; the quantitative values cited in this review (e.g., temperature-related shifts in protein and lipid fractions, or selection-related weight gains) should therefore be read as indicative magnitudes from individual studies rather than pooled or quality-weighted estimates. Future work could mitigate these limitations by pre-registering the review protocol (e.g., on OSF or PROSPERO) and applying an established risk-of-bias tool, further strengthening the transparency and reproducibility of the synthesis.

#### 5.5.2. Heterogeneity and Imbalance of the Available Data

The literature drawn upon is highly heterogeneous (species, substrates, rearing conditions, analytical methods), which limits the direct comparability of results. Moreover, the experimental data concentrate on a few model species (*T. molitor*, *H. illucens*, *A. domesticus*, *B. mori*), while most of the 41 recorded species remain poorly documented, only 7 of them being represented in the core corpus (Appendix A); general conclusions should therefore be extrapolated to all farmed species with caution.

#### 5.5.3. Language Bias and Publication Bias

Because it relied exclusively on English-language keywords, the search may have under-represented relevant work published in other languages. Despite the partial inclusion of grey literature via Google Scholar, a publication bias favoring positive results cannot be excluded, and some very recent publications or regulatory developments may have fallen outside the search period.

## 6. Conclusions

This critical review has analyzed, in an integrated manner, how rearing systems and factors influence the genetic, nutritional, and medicinal properties of edible insects. Rearing factors (nutritional, environmental, zootechnical, and biological) exert multidimensional and often interdependent effects on overall quality. Biochemical composition is not a fixed, species-intrinsic characteristic; it is the dynamic outcome of the interaction between the genetic makeup of the reared populations and the full set of production conditions to which they are subjected.

Conventional, semi-intensive, and industrial systems answer to distinct economic, technical, and cultural logics, and each carries specific advantages and limitations in terms of quality, cost, reproducibility, and sustainability. The growing industrialization of insect farming strengthens guarantees of standardization and sanitary quality, but in turn raises questions of sustainability, animal welfare, and economic accessibility that call for sustained scientific and regulatory vigilance. Ultimately, optimizing rearing practices is the central lever of an insect farming that is at once productive, high-quality, and sustainable. It offers not only a response to contemporary food challenges but also a rare opportunity to reinvent protein production in the service of ecological sustainability and global nutritional justice.

## Figures and Tables

**Table 1:** Comparison of edible insect rearing systems according to control level.

Rearing Factor	Conventional	Semi-Intensive	Intensive (Industrial)
Feed substrate	Local resources (residues, fruit, leaves)	Local resources and supplements (brans, oil cakes, by-products)	Formulated and balanced diets
Temperature	Uncontrolled (seasonal climate)	Partial control (shelters, cages)	Regulated within the optimal range per species
Relative humidity	Uncontrolled	Partial control	Precisely regulated
Ventilation and photoperiod	Natural	Simple devices, partial management	Artificial control
Density	Uncontrolled	Adjusted according to stage	Optimized per species and stage
Genetic selection	Absent (wild lines)	Empirical (retention of the best individuals)	Genetic/genomic selection programs
Microbiota	Variable	Partially stabilized	Controlled and stable
Sanitation and biosecurity	Rudimentary; contamination risk	Partial hygiene (cleaning, substrate management)	Strict and systematic (disinfection, microbiological control)
Technology and automation	Minimal	Simple tools	Automation and digital monitoring
Batch reproducibility	High variability	Intermediate	High
Set-up cost	Low	Moderate	High
Nutritional quality	Highly variable	Improved but variable	Stable and reproducible

Sources: van Huis et al. [2]; Rumpold & Schlüter [57]; Meyer-Rochow et al. [58]; Morales-Ramos et al. [44]; Eriksson & Picard [32]; Maciel-Vergara et al. [59].

**Table 3:** Strengths and weaknesses of edible insect rearing systems regarding the main production constraints.

Rearing System	Strengths	Weaknesses
Conventional	- Low set-up cost and minimal technological dependence - Valorization of local resources and by-products - High social acceptability where consumption is traditional (sub-Saharan Africa, Southeast Asia, Latin America)	- High variability in quality (biochemical composition) - Rudimentary biosecurity; high risk of microbiological contamination - No genetic selection (wild lines) - Low batch reproducibility; non-compliance with industrial-market requirements; limited traceability
Semi-intensive	- Favorable trade-off between cost and control of parameters - Partial control of temperature, humidity, density, and substrate - Improved quality and hygiene; accessible to small producers (service or cooperative models)	- Still-pronounced compositional variability - Empirical selection only; risk of inbreeding with a small breeding population - Incomplete standardization; intermediate sanitary guarantees
Intensive	- Stable, reproducible quality (standardized formulated diets) - Precise control of temperature, humidity, and density - Strict biosecurity (HACCP, substrate traceability) and regulatory compliance - Genetic and genomic selection programs; automation and digital monitoring	- High set-up and investment costs; technological dependence and skill requirements - Risk of genetic erosion and inbreeding in closed systems with small breeding populations - Reference genomic resources still insufficient for many species - Low social acceptability in Western contexts (neophobia, disgust)

Sources: Riekkinen et al. [37]; Sandrock et al. [128]; Hansen et al. [84]; Lahteenmaki-Uutela et al. [162]; Kroger et al. [169]; Ros-Baro et al. [166]; Tanga & Kababu [167].

## Data Availability

No new data were created or analyzed in this study. Data sharing is not applicable to this article.

## Abbreviations

The following abbreviations are used in this manuscript:

AdDV	Acheta domesticus densovirus
CAT	Catalase
COX	Cyclooxygenase
DM	Dry matter
DNA	Deoxyribonucleic acid
Dnmt3	DNA methyltransferase 3
DPP-IV	Dipeptidyl peptidase-IV
EAAI	Essential amino acid index
EFSA	European Food Safety Authority
FAO	Food and Agriculture Organization of the United Nations
GWAS	Genome-wide association study
HACCP	Hazard Analysis and Critical Control Points
HSP70	Heat-shock protein 70
HSP90	Heat-shock protein 90
LOX	Lipoxygenase
OSF	Open Science Framework
PROSPERO	International Prospective Register of Systematic Reviews
PUFAs	Polyunsaturated fatty acids
RH	Relative humidity
ROBINS	Risk Of Bias In Non-randomized Studies
SNP	Single-nucleotide polymorphism
SOD	Superoxide dismutase
SYRCLE	Systematic Review Centre for Laboratory Animal Experimentation
UV	Ultraviolet

## Appendix A

**Table A1:** Main farmed/reared edible insect species (n = 41), listed in taxonomic order, with their representation in the 96-study core corpus.

Order	Family	Scientific Name	Common Name	Consumed Stage	Rearing Type	Rearing Regions (Countries)	Main Use	References	Core Corpus (Yes/No)
**Blattodea**	Blaberidae	*Blaptica dubia* (Serville, 1838)	Dubia roach	Nymphs and adults	Semi-industrial and artisanal	Worldwide	Animal feed	Yee et al. [184]; Barras [185]	No
		*Gromphadorhina portentosa* (Schaum, 1853)	Madagascar hissing cockroach	Nymphs and adults	Artisanal	Worldwide	Animal feed	Carvalho et al. [186]	No
	Blattidae	*Periplaneta americana* (Linnaeus, 1758)	American cockroach	Nymphs and adults	Industrial and semi-industrial	Asia (China)	Traditional medicine and animal feed	Chen et al. [187]	No
**Orthoptera**	Acrididae	*Locusta migratoria* (Linnaeus, 1758)	Migratory locust	Adult and nymph	Industrial	Europe (Netherlands)	Human and animal food	van Huis et al. [2]	Yes
		*Patanga succincta* (Johannson, 1763)	Bombay locust	Adult	Semi-industrial and artisanal	Southeast Asia	Human food	Dobermann et al. [54]	No
	Gryllidae	*Acheta domesticus* (Linnaeus, 1758)	House cricket	Adult	Industrial, semi-industrial and artisanal	Worldwide	Human and animal food	European Union [188]	Yes
		*Gryllodes sigillatus* (Walker, 1869)	Banded cricket	Adult	Industrial, semi-industrial and artisanal	Worldwide	Human and animal food	European Union [188]	No
		*Gryllus assimilis* (Fabricius, 1775)	Jamaican field cricket	Adult	Industrial, semi-industrial and artisanal	America, Europe	Animal feed	European Union [188]	No
		*Gryllus bimaculatus* De Geer, 1773	Field cricket	Adult	Industrial and semi-industrial	Asia (Thailand, China, Korea), Africa, Europe	Human and animal food	Magara et al. [189]	Yes
		*Teleogryllus occipitalis* (Serville, 1838)	Cricket	Adult	Semi-industrial and artisanal	Southeast Asia (Thailand, Laos)	Human food	Magara et al. [189]	No
**Hemiptera**	Coccidae	*Ericerus pela* (Chavannes, 1848)	Chinese white wax scale	Larva	Semi-industrial and artisanal	Asia (China, Japan, Korea)	Insect wax	Yang et al. [190]	No
	Dactylopiidae	*Dactylopius coccus* Costa, 1835	Cochineal	Adult	Industrial, semi-industrial and artisanal	America (Peru, Mexico), Canary Islands	Dye (carmine/carminic acid)	Chavez-Moreno et al. [191]	No
	Kerriidae	*Kerria lacca* (Kerr, 1782)	Lac insect	Larva/adult	Semi-industrial and artisanal	Asia (India, Thailand)	Shellac, dye	Sharma [192]	No
**Coleoptera**	Curculionidae	*Rhynchophorus ferrugineus* (Olivier, 1790)	Red palm weevil	Larva	Semi-industrial and artisanal	Asia (Indonesia, Malaysia, Papua New Guinea and Thailand)	Human food	Chinarak et al. [193]	No
		*Rhynchophorus palmarum* (Linnaeus, 1758)	South American palm weevil	Larva	Semi-industrial and artisanal	South America (Venezuela, Brazil, Peru, Paraguay,)	Human food	Jaramillo-Vivanco et al. [194]	No
		*Rhynchophorus phoenicis* (Fabricius, 1801)	African palm weevil	Larva	Semi-industrial and artisanal	Africa (Ghana)	Human food	Debrah et al. [195]	No
	Cerambycidae	*Thysia wallichii* (Hope, 1831)	Longhorn beetle	Larva	Artisanal	Asia (India)	Human food	Kiewhuo et al. [62]	No
	Scarabaeidae	*Protaetia brevitarsis* (Lewis, 1879)	White-spotted flower chafer	Larva	Industrial and semi-industrial	East Asia (South Korea, China)	Human food and traditional medicine	Ham et al. [196]	No
	Tenebrionidae	*Alphitobius diaperinus* (Panzer, 1797)	Buffalo worm	Larva	Industrial	Europe (Netherlands, Belgium)	Animal feed	European Union [197]	No
		*Tenebrio molitor* Linnaeus, 1758	Yellow mealworm	Larva	Industrial, semi-industrial and artisanal	Worldwide	Human and animal food	EFSA NDA Panel [40]	Yes
		*Tenebrio obscurus* Fabricius, 1792	Dark mealworm	Larva	Industrial and semi-industrial	Europe, North America, South Asia	Animal and human food	Bai et al. [198]	No
		*Zophobas morio* (Fabricius, 1776)	Superworm	Larva	Industrial and semi-industrial	Europe, America, Southeast Asia	Animal and human food	Pumnuan et al. [199]	No
**Lepidoptera**	Bombycidae	*Bombyx mori* (Linnaeus, 1758)	Domestic silkworm	Larva/pupa	Industrial sericulture	Asia (China, India)	Silk production	Xia et al. [200]	Yes
	Crambidae	*Omphisa fuscidentalis* (Hampson, 1896)	Bamboo worm	Larva	Artisanal	Asia (Thailand, Laos, Myanmar, China)	Human food	Thapa [201]	No
	Pyralidae	*Galleria mellonella* (Linnaeus, 1758)	Wax moth	Larva	Industrial, semi-industrial and artisanal	Europe, America	Animal feed	Francardi et al. [202]	No
	Saturniidae	*Antheraea assamensis* (Helfer, 1837)	Muga silkworm	Larva/pupa	Semi-industrial and artisanal sericulture	Asia (India)	Silk production	Das [203]	No
		*Antheraea pernyi* (Guérin-Méneville, 1855)	Chinese oak silkworm	Larva/pupa	Semi-industrial and artisanal sericulture	Asia (China, Korea)	Silk production	Li et al. [204]	No
		*Antheraea yamamai* (Guérin-Méneville, 1861)	Japanese oak silkworm	Larva/pupa	Artisanal sericulture	Asia (Japan)	Silk production	Kim et al. [205]	No
		*Samia ricini* (Donovan, 1798)	Eri silkworm	Larva/pupa	Industrial sericulture	Asia (India)	Silk production	Kandasamy et al. [206]	No
**Hymenoptera**	Apidae	*Apis cerana* Fabricius, 1793	Asian honeybee	Brood	Industrial, semi-industrial and artisanal Beekeeping	Asia (India, China, Nepal, Thailand, Vietnam)	Hive products	Qu et al. [207]	No
		*Apis mellifera* Linnaeus, 1758	Western honeybee	Brood	Industrial beekeeping	Worldwide	Hive products	FAO et al. [39]	Yes
		*Heterotrigona itama* (Cockerell, 1918)	Stingless bee	Brood	Semi-industrial meliponiculture	Southeast Asia (Malaysia, Indonesia)	Hive products	Wan et al. [208]	No
		*Melipona beecheii* Bennett, 1831	Maya stingless bee	Brood	Artisanal meliponiculture	Central America (Mexico)	Hive products	Villanueva-G et al. [209]	No
		*Scaptotrigona bipunctata* (Lepeletier, 1836)	Stingless bee	Brood	Artisanal meliponiculture	South America (Brazil, Argentina, Paraguay)	Hive products	Martínez-Fortun et al. [210]	No
		*Scaptotrigona depilis* (Moure, 1942)	Stingless bee	Brood	Artisanal meliponiculture	South America (Brazil)	Hive products	Macêdo Rocha et al. [211]	No
		*Scaptotrigona mexicana* (Guérin-Méneville, 1845)	Stingless bee	Brood	Semi-industrial meliponiculture	North and Central America	Hive products	Gerginova et al. [212]	No
		*Scaptotrigona pectoralis* (Dalla Torre, 1896)	Stingless bee	Brood	Artisanal meliponiculture	Central America (Mexico, Belize,)	Hive products	Reyes-González et al. [213]	No
		*Scaptotrigona postica* (Latreille, 1807)	Stingless bee	Brood	Artisanal meliponiculture	South America (Brazil, Amazonia)	Hive products	Leao et al. [214]	No
		*Tetragonula laeviceps* (Smith, 1857)	Stingless bee	Brood	Semi-industrial meliponiculture	Asia (Thailand, Malaysia, Indonesia)	Hive products	Chuttong et al. [215]	No
**Diptera**	Muscidae	*Musca domestica* Linnaeus, 1758	House fly	Larva	Industrial and artisanal	Worldwide	Animal feed	European Union [188]	No
	Stratiomyidae	*Hermetia illucens* (Linnaeus, 1758)	Black soldier fly	Larva	Industrial, semi-industrial and artisanal	Worldwide	Animal feed	European Union [188]	Yes

**Note:** Species are listed by taxonomic order following the systematic sequence, then alphabetically by family and by species. Unless otherwise indicated, all species appear in Jongema’s [46] world list of edible insects. Three taxa are listed only at genus level (*Gromphadorhina* sp., *Protaetia* sp. and *Gryllodes* sp.) the genus being recorded as edible without the specific species being cited. Five species do not appear in Jongema [46] and their edibility relies on specific references: *Alphitobius diaperinus* (European Union [197]), *Tenebrio obscurus* (Bai et al. [198]), *Tetragonula laeviceps* (Chuttong et al. [215]), *Heterotrigona itama* (Wan et al. [208]) and *Blaptica dubia* (Yee et al. [184]; Barras [185]). Core corpus: “Yes” indicates that at least one study from the 96-study core corpus reporting effects of rearing conditions on the species is synthesized in Section 4 or Table 2; “No” indicates that the species is listed for inventory purposes only.
